# Upper versus lower airway microbiome and metagenome in children with cystic fibrosis and their correlation with lung inflammation

**DOI:** 10.1371/journal.pone.0222323

**Published:** 2019-09-19

**Authors:** Mariana E. Kirst, Dawn Baker, Eric Li, Mutasim Abu-Hasan, Gary P. Wang

**Affiliations:** 1 Department of Medicine, Division of Infectious Diseases and Global Medicine, University of Florida College of Medicine, Gainesville, FL, United States of America; 2 Department of Pediatrics, Division of Pediatric Pulmonology, University of Florida College of Medicine, Gainesville, FL, United States of America; 3 Medical Service, Infectious Disease Section, North Florida/South Georgia Veterans Health System, Gainesville, FL, United States of America; Laurentian, CANADA

## Abstract

**Objective:**

Airways of children with cystic fibrosis (CF) harbor complex polymicrobial communities which correlates with pulmonary disease progression and use of antibiotics. Throat swabs are widely used in young CF children as a surrogate to detect potentially pathogenic microorganisms in lower airways. However, the relationship between upper and lower airway microbial communities remains poorly understood. This study aims to determine (1) to what extent oropharyngeal microbiome resembles the lung microbiome in CF children and (2) if lung microbiome composition correlates with airway inflammation.

**Method:**

Throat swabs and bronchoalveolar lavage (BAL) were obtained concurrently from 21 CF children and 26 disease controls. Oropharyngeal and lung microbiota were analyzed using 16S rRNA deep sequencing and correlated with neutrophil counts in BAL and antibiotic exposure.

**Results:**

Oropharyngeal microbial communities clustered separately from lung communities and had higher microbial diversity (p < 0.001). CF microbiome differed significantly from non-CF controls, with a higher abundance of *Proteobacteria* in both upper and lower CF airways. Neutrophil count in the BAL correlated negatively with the diversity but not richness of the lung microbiome. In CF children, microbial genes involved in bacterial motility proteins, two-component system, flagella assembly, and secretion system were enriched in both oropharyngeal and lung microbiome, whereas genes associated with synthesis and metabolism of nucleic acids and protein dominated the non-CF controls.

**Conclusions:**

This study identified a unique microbial profile with altered microbial diversity and metabolic functions in CF airways which is significantly affected by airway inflammation. These results highlight the limitations of using throat swabs as a surrogate to study lower airway microbiome and metagenome in CF children.

## Introduction

Cystic Fibrosis (CF) is the most common life-shortening autosomal recessive disorder in persons of European ancestry, affecting more than 30,000 Americans with an estimated 1,000 new cases diagnosed in the US each year [1]. It is a multisystem disease caused by mutations in Cystic Fibrosis Transport Regulator (CFTR) gene, which encodes a chloride/bicarbonate channel located at the cell membrane of different cells including the airway epithelium and bronchial glands. Defective CFTR in the airways results in dehydration of secretions and defective innate immunity, allowing airway colonization of pathogenic bacteria leading to a relentless cycle of infection, inflammation and irreversible destruction of the airways [2]. Despite advances in the clinical management of CF, progressive lung disease remains the primary cause of morbidity and mortality [3].

It has long been appreciated that a diverse set of organisms colonizes the CF lung. Growing evidence suggests that bacteria colonize the airways of most CF patients within the first year of life, and colonization or co-infection involving different species of bacteria is common [4–7]. Historically, only a handful of organisms have been implicated as clinically relevant in CF respiratory infections, which include *Pseudomonas aeruginosa*, *Staphylococcus aureus*, *Haemophilus influenzae*, and the *Burkholderia cepacia complex* ^*8*^. The pathogenic roles of other organisms, such as *Stenotrophomonas maltophila*, *Achromobacter xylosoxidans*, or nontuberculoous mycobacteria, are less clear [8–19]. Nevertheless, once bacteria colonize the CF lung, a repeated cycle of infection, inflammation, and lung damage occurs. Recent advances in non-culture based methods have provided new insights into the CF airway microbiome, raising the questions of potential roles of microorganisms not easily detectable by culture-based methods and the role of relative richness and diversity of different microbial communities in airway inflammation and disease progression. Thus, a detailed understanding of microbial environment in CF airways and its association with airway inflammation is critical for understanding disease pathogenesis and developing new strategies to prevent or treat infectious complications.

Airway microbiota in adults with CF is generally assessed using sputum samples. However, routine culture targets only a limited number of pathogens such as *Pseudomonas aeruginosa*, *Staphylococcus aureus*, *Haemophilus influenzae*, *Achromobacter and Burkholderia* [20–22]. For the past decade, lung microbiome of CF patients has been examined using culture-independent methods that utilize phylogenetic information derived from bacterial 16S rRNA gene sequences [23–27]. Results from these studies demonstrate that CF lung is colonized by a large group of bacteria, more diverse than previously thought. Examinations of microbiome in sputum, bronchioalveolar lavage, and throat swabs in CF patients have revealed detectable differences in bacterial profile in each environment [28–30]. In infants and younger children, it is often difficult to obtain sputum samples because of their difficulties in cooperating or because of decreased mucus production at early stages of lung disease. Therefore, throat swabs, which can be easily obtained in the youngest population, are widely used as a surrogate for detecting potentially pathogenic microorganisms that may be present in lower airways. However, studies comparing throat swabs with lower airway cultures using culture-based methods have shown variable sensitivity and specificity when compared to bronchoalveolar lavage (BAL) culture, the gold standard [31–34]. Thus, the relationship between upper and lower airway microbial communities remains poorly understood. It is not known to what extent oropharyngeal microbial communities sampled using throat swabs resemble the microbial populations in the lungs of CF children.

The objective of this study was to compare oropharyngeal and lung microbiome sampled concurrently using throat swabs and BAL, and examine the relationship between lung microbiome and severity of airway inflammation and antibiotic exposure. Using 16S rRNA sequencing and PICRUSt [35] (a bioinformatics tool for predicting metagenome functional content from marker gene surveys), we showed that oropharyngeal microbiome of CF children do not reflect the microbial composition and community structure of their lung microbiota, and that CF airway microbiome and its predictive functions differ significantly from that of non-CF controls.

## Materials and methods

### Study population

Children with or without CF who underwent clinically indicated bronchoscopy and BAL were recruited for the study between June 2012 and November 2013. The clinical indication for bronchoscopy in CF patients was to evaluate airway inflammation and mucus plugging and to obtain brochoalveolar lavage culture to guide antibiotic therapy. The indication for bronchoscopy in non-CF patients was chronic cough and/or wheezing of unknown etiology. At the time of bronchoscopy, some of the CF subjects were hospitalized and were receiving IV antibiotics, and others were on oral antibiotics or have been recently treated with antibiotics. Some subjects had no recent exposure to antibiotics. Informed consent was obtained from all patients or their parents (depending on patient’s age) for study participation and procedures. Electronic medical records were reviewed to evaluate disease severity. The study was approved by the Institutional Review Board at the University of Florida.

### Bronchoscopy and specimen collection

Although older children could expectorate to produce sputum, throat swab was used in all subjects for consistency because it could be applied to the entire age range of all subjects. Throat swabs were obtained immediately prior to bronchoscopy from all patients by passing two cotton swabs across the posterior pharynx simultaneously. One sample swab was sent for routine bacterial culture and susceptibility testing, and the other was frozen for subsequent microbiome analysis. Flexible bronchoscopy was performed in the majority of patients in a bronchoscopy suite under conscious sedation. After a patient was adequately sedated, a bronchoscope was passed through right or left nasopharynx to oropharynx to examine the glottic structures. Topical 1% lidocaine was then instilled on vocal cords before a scope was passed though vocal cords to examine the entire tracheobronchial tree. BAL was obtained from the pulmonary lobe of interest based on radiological and/or bronchoscopic findings. BAL was performed by injecting three separate aliquots of normal saline (1 ml/kg) after wedging in a lobar or segmental bronchus and suctioning the aliquot into a trap cup. To minimize contamination from the upper airway, suctioning of upper airways was avoided to prevent entry of upper airway microorganisms into the suctioning channel before entering the lower airways. In addition, tight wedging of the scope in the lower airways was performed before instilling saline to avoid contaminating the surface of the scope with microorganisms that may have been picked up from the upper airways. For a few subjects, the bronchoscopy procedure was done in the operating room under general anesthesia due to patient-related risks. In these instances, a bronchoscope was passed through an endotracheal tube or a laryngeal mask, and BAL was obtained as described above.

### Sample analysis

DNA was extracted from throat swab or BAL samples using the MoBio PowerSoil kit according to manufacturers’ instructions. Water and DNA extraction kits were used as negative controls for background 16S rRNA contamination. Bronchoalveolar lavage fluid (BAL) was centrifuged at 300 g for 10 minutes to remove human cells or cell debris prior to DNA extraction. To extract DNA from swab samples, swabs were immersed in 1x PBS, and then underwent further processing. Due to low DNA concentrations, a 2-step nested PCR was performed. Purified DNA was amplified using 16S rRNA primers 8F and 1452R, followed by amplification of the V1-V3 hypervariable region of 16S rRNA gene using barcoded primers. Amplification of the expected size was confirmed by agarose gel electrophoresis, and bands were excised and purified by gel extraction (Qiagen, Valencia, CA). DNA concentration was measured using Qubit (Invitrogen, Carlsbad, CA), and samples were pooled at an equimolar concentration and deep sequenced using the Illumina MiSeq platform (Illumina, San Diego, CA).

### Bioinformatic analysis

Raw paired-end reads generated from Illumina MiSeq runs were processed using custom scripts written in R [36] for de-multiplexing, quality filtering, and trimming reads. Reads were filtered based on exact matches to the barcode and primer sequences with an average quality score of 30 or higher. Samples were de-multiplexed according to the combination of unique variable length barcodes (4 to 8 nt) on each paired end. In downstream analysis, barcodes and primers were trimmed. To reconstruct the original contiguous amplicon, paired end reads were joined using FLASh [37], with a minimum of 10 bp overlap. USEARCH alignment was employed with a minimum of 97% identity and 50% aligned query threshold to assign reference OTUs with taxonomic information from the Silva database to each joined read [38,39]. Reads that did not meet the filtering criteria were excluded from subsequent analysis. Rarefaction for diversity analyses was performed at an even sub-sampling depth of 15,000 sequences at 10 iterations per sample using scripts from QIIME (version 1.8.0). OTUs with 1–2 reads and six samples that did not meet the minimum required depth were removed. Sub-tables were created based on taxonomic levels and reads that aligned to negative controls were removed from subsequent analysis. The OTU table was normalized using the Wisconsin command in the vegan package. Additional statistical analyses were performed using LEfSe [40] and in R to determine differentially significant features between groups. Subsequent automated analyses, including alpha diversity measures, were calculated using formulas and were generated in R. Beta diversity analysis was carried out in QIIME (version 1.8.0) using Unifrac distance metric [41,42]. Statistical significance of clustering by group was determined with Permutational Multivariate Analysis of Variance (PERMANOVA). Prediction of metagenome functional content was performed based on 16S rRNA sequences using PICRUSt (phylogenetic investigation of communities by reconstruction of unobserved states) [35].

## Results

### Subjects and samples analyzed

To compare oropharyngeal and lung microbiome, we sampled the oropharynx and the lungs of 21 CF and 26 non-CF children (disease control) using throat swabs and BAL, respectively. Clinical characteristics of all subjects are shown in Table 1. The median age of CF children was higher compared to non-CF controls (8 vs 2.5 years of age; p = 0.007; Table 1). As expected, 14 of 20 (70%) BAL samples from CF children had high levels of neutrophilic inflammation, compared to 7 of 25 (28%) in non-CF controls (p = 007). *Pseudomonas aeruginosa* was cultured in 4 of 18 (22%) throat swabs and 2 of 21 (9.5%) BAL samples from CF children, compared to none from throat swabs or BAL cultures in non-CF controls. Similarly, *S*. *aureus* was isolated from 5 of 18 (28%) throat swabs and 8 of 21 BAL samples (38%) from CF children, compared to 2 of 24 (8%) throat swabs and 3 pf 26 (12%) BAL from non-CF controls. 8 of 21 CF children (38%) received concurrent antibiotics at the time of sampling and 13 of 20 (65%) had antibiotic exposure within 1 month prior to sampling, compared to 3 of 25 (12%) and 6 of 26 (23%), respectively, in non-CF controls (p = 0.08 and 0.007, respectively). No CF subjects received CFTR modulator therapy.

**Table 1:** Clinical characteristics of subjects in this study.

		CF^#^	non-CF controls^#^	p value *
		n = 21	n = 26	
Median Age (quartile)	Age	8 (3, 13)	2.5 (1.4, 6.8)	0.007
Gender, n (%)	Male	14	16	0.768
Inflammation based on bronchoscopy	Mild or no Inflammation	10/21, 48%	20/26, 77%	0.0659
	Moderate/Severe	11/21, 52%	6/26, 23%	0.0659
BAL neutrophils	>40%	14/20, 70%	7/25, 28%	0.007
BAL culture	Pseudomonas aeruginosa (n, %)	2/21, 9.5%	0/26, 0%	0.19
	S. aureus (n, %)	8/21, 38%	3/26, 12%	0.043
	Steno. maltophila or Pseudo. alcaligenes (n, %)	3/21, 14%	0/26, 0%	0.08
	H. influenzae (n, %)	4/21, 19%	5/26, 19%	1
	M. catarrhalis (n, %)	2/21, 10%	8/26, 31%	0.15
Throat Swab culture	Pseudomonas aeruginosa (n, %)	4/18, 22%	0/24, 0%	0.027
	S. aureus (n, %)	5/18, 28%	2/24, 8%	0.12
Viral PCR	Positive (n, %)	7/17, 41%	11/26, 42%	1
Antibiotics exposure ^	at the time of sampling	8/21, 38%	3/25, 12%	0.08
	within the previous month	13/20, 65%	6/26, 23%	0.007
PFTs	FEV1 < 80% predicted	9/15, 60%	4/9, 44%	0.675

* P value is based on Fisher’s exact test for the categorical variables, and 2-sample t test for the continuous variables

# Denominator indicates the total number of subjects with available data

^ Any oral, inhaled, or intravenous antibiotics

Amplification of 16S rRNA gene segment was successful for 19 throat swabs and 18 BAL samples from CF patients and 23 throat swabs and 25 BAL samples from non-CF disease controls. The failure rate for amplification was comparable to a prior study [43]. Illumina sequencing of these 37 CF and 48 non-CF specimens yielded a total of 4,022,222 reads (median: 31,680; range: 3,260–323,494) spanning the V1-V3 hypervariable region of the bacterial 16S rRNA gene.

### Microbial diversity and community structure of oropharyngeal and lung microbiome

**Fig 1:**
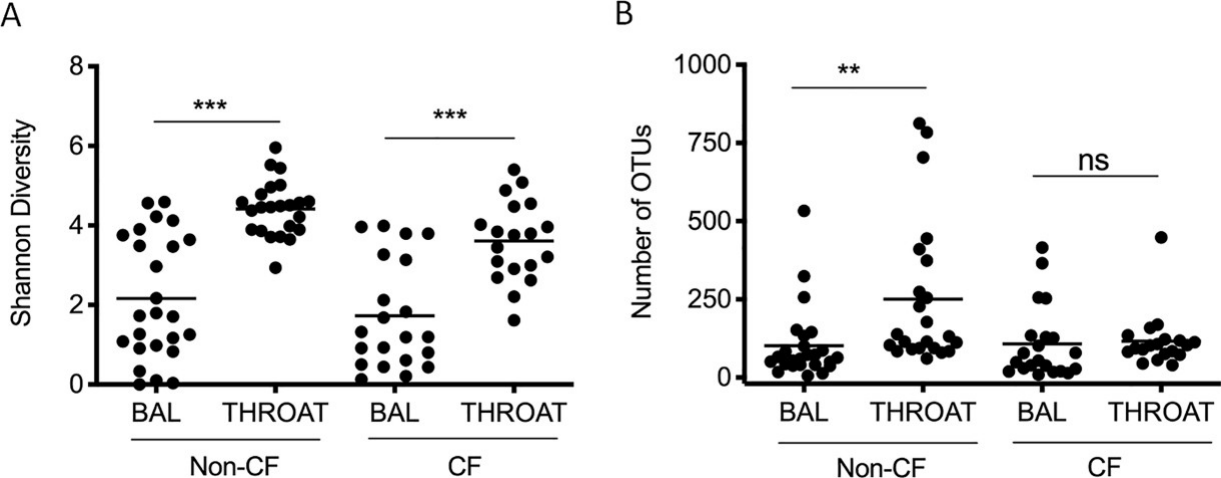
Microbial diversity and species richness of upper and lower airway microbiome in CF children and non-CF disease controls. (A) Shannon indices were calculated to denote microbial diversity and are plotted on the y-axis. Upper airway microbiome was sampled using throat swab, and lower airway microbiome was sampled by bronchioalveolar lavage (BAL). Values were compared using unpaired t-test. (B) The number of OTUs for each sample is plotted on the y-axis. CF = cystic fibrosis. Non-CF denotes non-CF disease controls. *** p < 0.001, ** p<0.01, ns = not significant.

**Fig 2:**
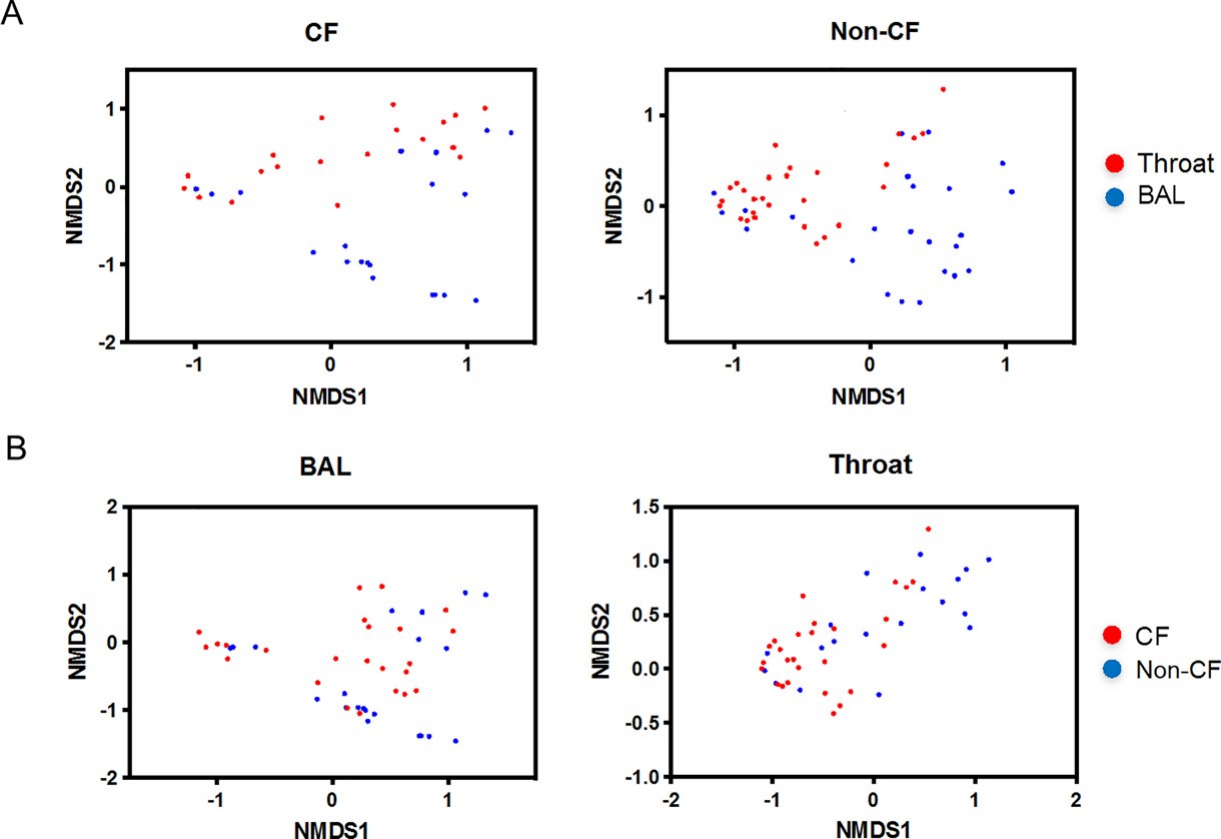
Plots showing clustering of airway microbiome communities. (A) Upper airway and lower airway microbiome of CF (left) and non-CF (right) children represented by nonmetric multidimensional scaling (NMDS) of pairwise weighted Unifrac distances. Weighted UniFrac distance matrices were generated using QIIME v 1.8.0 and visualized via NMDS in the R statistical environment using the *vegan* package. (B) Lower (left) and upper airway (right) microbial communities stratified by CF and non-CF status represented by NMDS of pairwise weighted Unifrac distances. Statistical significance of clustering by group was determined using PERMANOVA.

The microbial diversity (Fig 1A) was significantly higher in the oropharyngeal microbiome (Throat) compared to the lung microbiome (BAL) in both CF and non-CF children. Species richness (Fig 1B) was significantly higher in the oropharyngeal microbiome compared to the lung microbiome in non-CF controls but not CF children. Compared to non-CF controls, the airway microbiome of CF children (both Throat and BAL) had lower microbial diversity (Fig 1A). However, these differences were not statistically significant. Non-metric multi-dimentional scaling ordination analysis (Fig 2A) showed significant separation between oropharyngeal (Throat) and lung microbial communities (BAL) in both CF and non-CF controls (p = 0.001 and p = 0.001, respectively, PERMANOVA), indicating that oropharyngeal microbiome does not resemble the lung microbiome. Moreover, both oropharyngeal and lung microbiome differed significantly between CF and non-CF controls (Fig 2B; Throat: p = 0.004; BAL: p = 0.013; PERMANOVA), suggesting a unique airway microbiome in cystic fibrosis.

### Microbial composition of CF and non-CF airway microbiome

**Fig 3:**
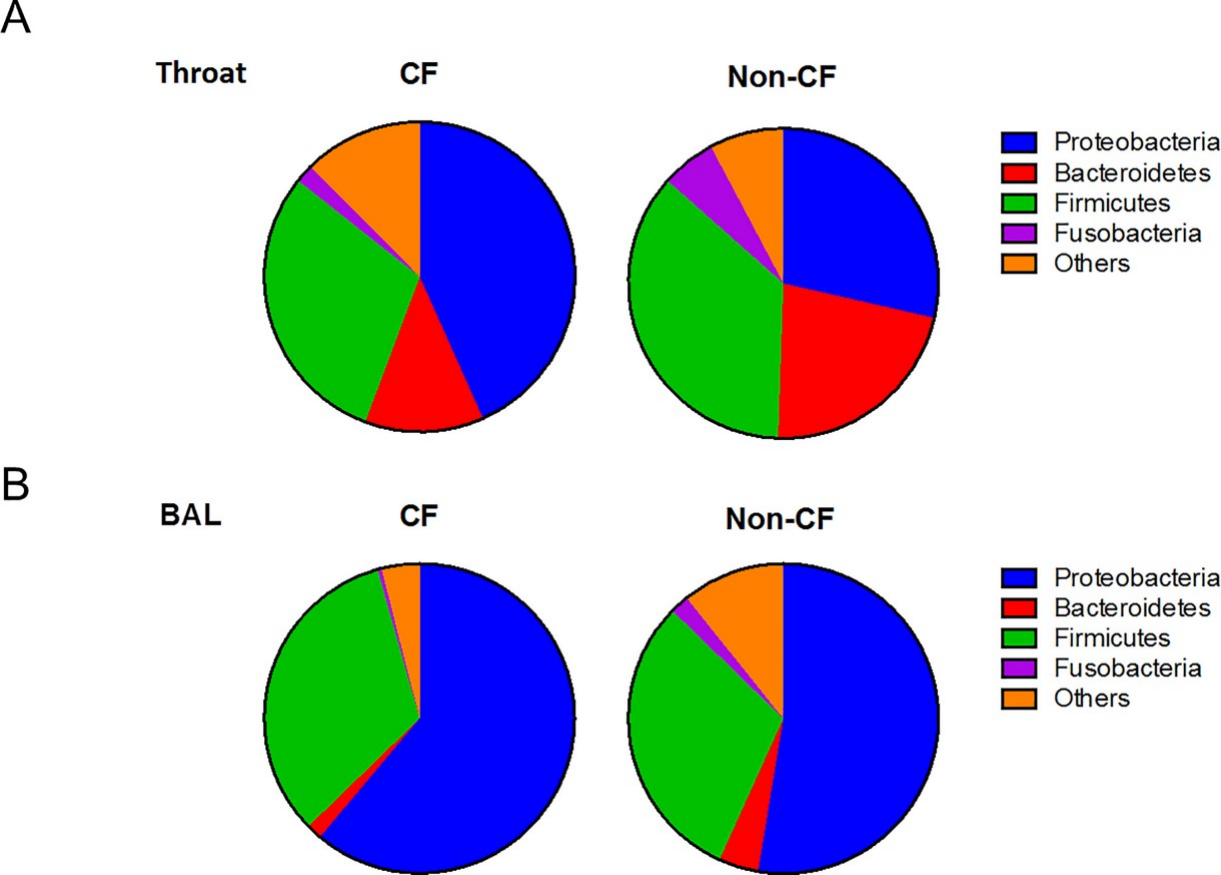
Relative proportions of microbial taxa of the aggregate microbiome at the phylum level. (A) microbial composition of the upper airways of CF children (left) and non-CF controls (right), (B) microbial composition of the lower airways of CF children (left) and non-CF controls (right). Each color denotes the proportion of taxa at the level of the phylum indicated.

**Fig 4:**
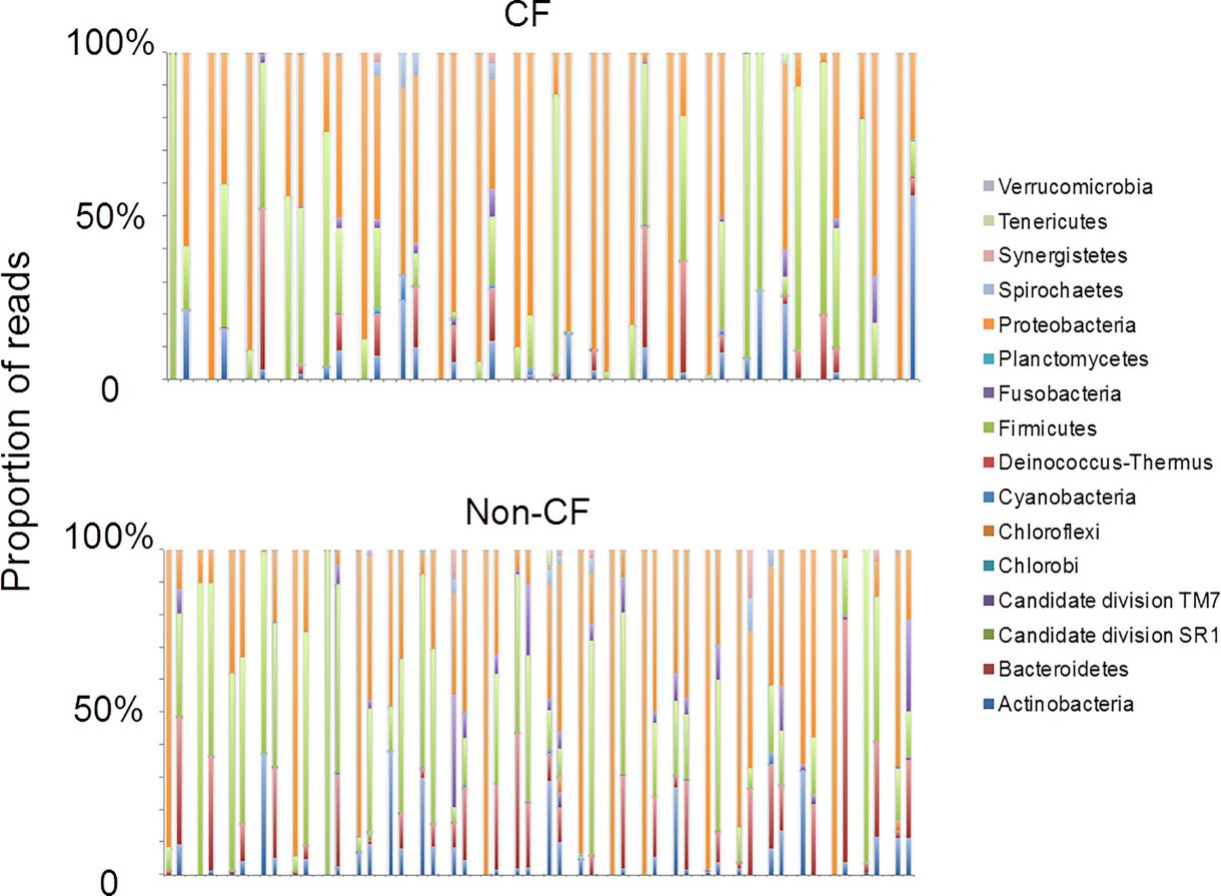
Phylum level composition of upper and lower airway microbiome sampled concurrently in each subject. Proportions of microbial taxa are shown on the y-axis, and paired upper and lower airway samples are shown side by side (left: BAL; right: Throat) for comparison. Top: CF children; bottom: non-CF controls.

We next compared the composition and distribution of bacterial taxa in the airway microbiome. The oropharyngeal communities of both CF and non-CF controls were dominated by *Proteobacteria* and *Firmicutes*, and to a lesser extent *Bacteroidetes* (Fig 3A). In the lung, *Proteobacteria* and *Firmicutes* also accounted for the vast majority of the lung microbial population, but *Bacteroidetes* were a minority population (Fig 3B). Although the aggregate microbiome between upper and lower airways appeared similar (Fig 3), the concordance between paired upper and lower airway microbiome within subjects (r>0.3; Spearman Correlation) was low, which was observed in only 4/20 (20%) CF and 7/ 24 (29%) non-CF children (Fig 4).

**Fig 5:**
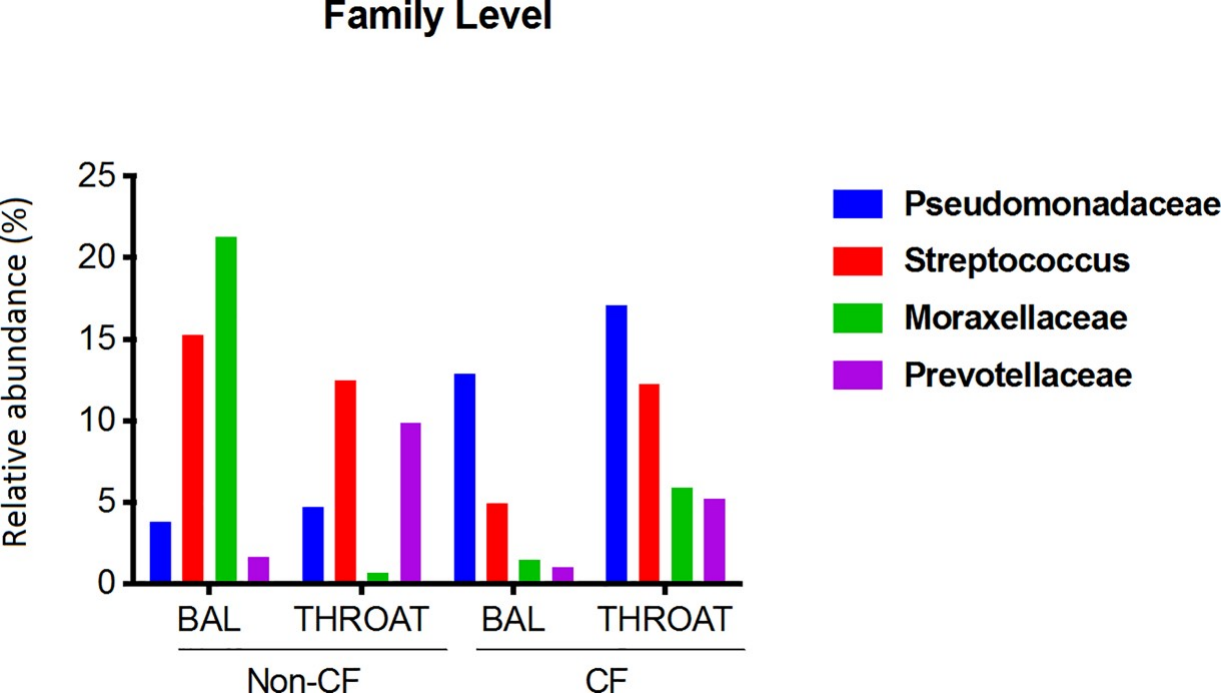
Relative abundance of major bacterial taxa at the family level in upper and lower airways of CF and non-CF children. Proportion of reads for each family is shown on the y-axis.

Compared to non-CF controls, a higher abundance of *Proteobacteria* was observed in both upper and lower airway microbiota of CF children. While members of *Streptococcaceae* were prevalent in the oropharyngeal microbiome of all subjects, *Pseudomonadaceae* were more abundant in CF (Fig 5). *Pseudomonadaceae* were readily detectable in both upper and lower airways of CF (17% and 13%, respectively). Interestingly, *Moraxellaceae* dominated the lung microbiome of non-CF controls, which accounted for approximately 21% of the microbial community. In contrast, *Moraxellaceae* constituted only 1% of the CF lung microbiome.

### Functional profiling of airway microbial communities

**Fig 6:**
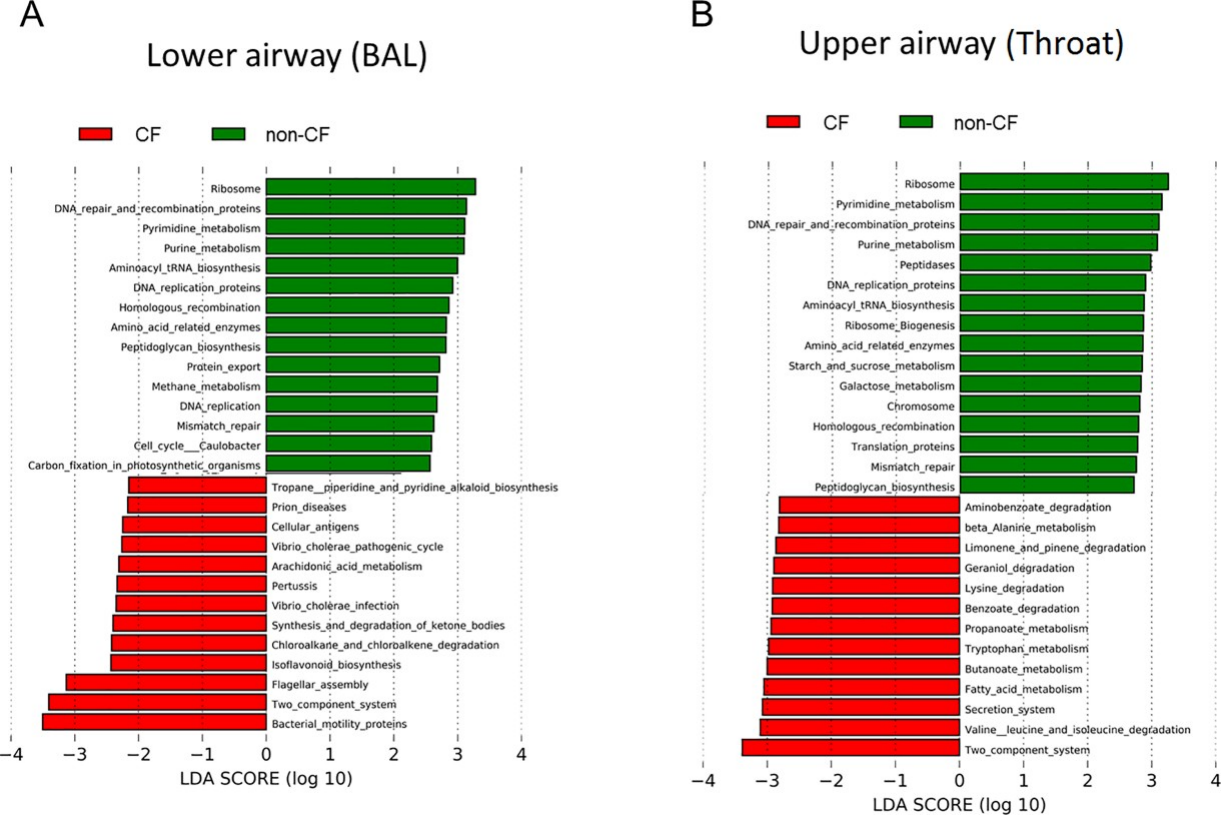
**Differentially abundant gene functions comparing CF and non-CF metagenomes in (A) lower and (B) upper airways.** Functional categories of genes of the airway metagenome were predicted using PICRUSt, and differentially abundant functions were then identified using linear discriminant analysis (LDA) coupled with effect size measurements (LEfSe). Gene functions enriched in non-CF metagenome are indicated with positive linear discriminant analysis scores (green), and functions differentially enriched in CF metagenome are indicated with negative linear discriminant analysis scores (red).

Compared to non-CF controls, genes associated with bacterial motility proteins, two-component system, flagella assembly were significantly more abundant in the CF BAL metagenome (Fig 6A), and genes associated with two component system, valine leucine and isoleucine degradation, and secretion system were differentially enriched in the CF oropharynx metagenome (Fig 6B). For both CF and non-CF controls, upper airway metagenomes were enriched with genes involved in amino sugar and nucleotide sugar metabolism, fructose and mannose metabolism, galactose metabolism, starch and sucrose metabolism, and phosphotransferase system, whereas genes associated with amino acid biosynthesis, degradation and metabolism, and fatty acid biosynthesis were overrepresented in lower airway metagenomes (S1 Fig).

### Lung microbiome and airway inflammation

**Fig 7:**
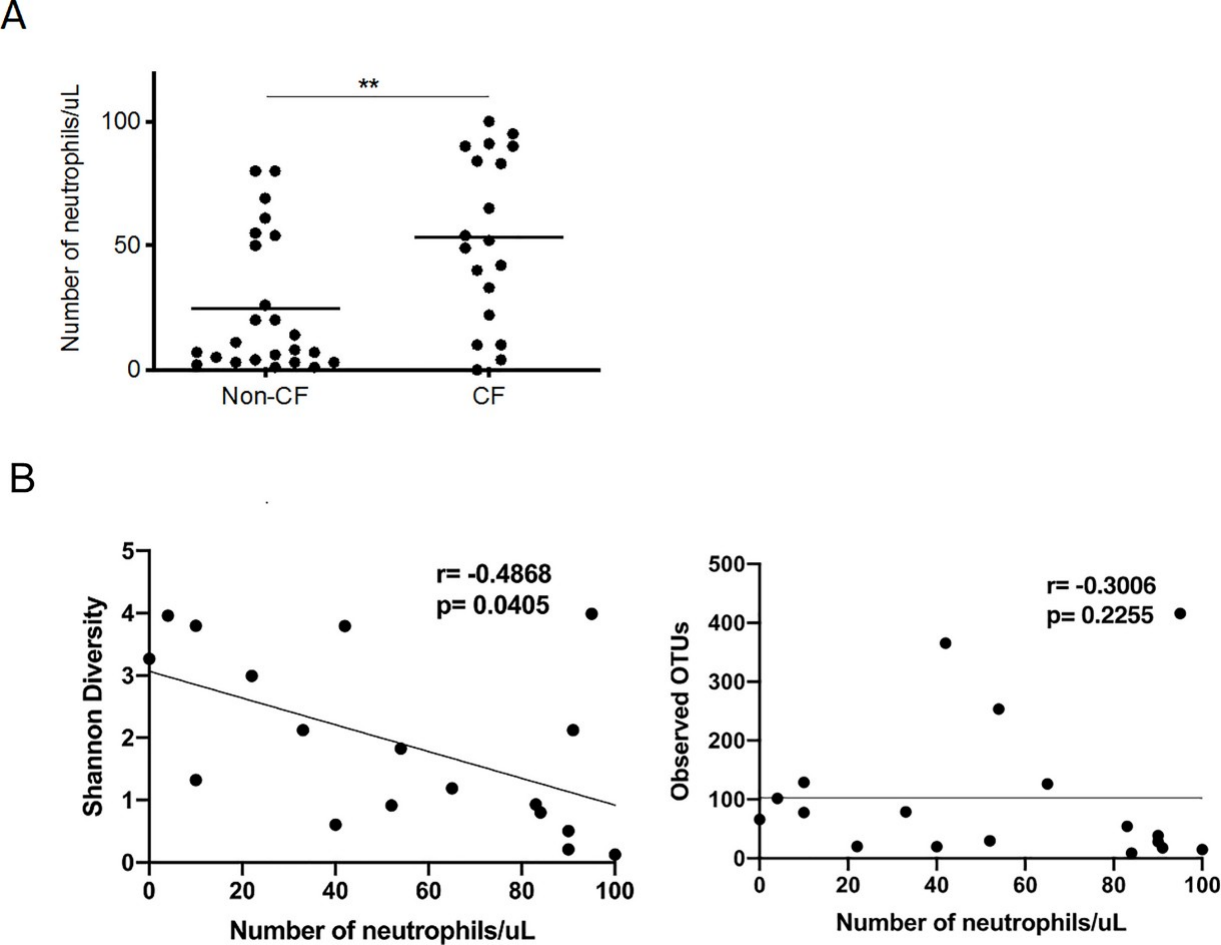
Lung microbiome diversity and inflammation in CF and non-CF controls. (A) Neutrophil counts in CF and non-CF controls. Neutrophil count in the BAL was used as a surrogate for lung inflammation, and is shown in the y-axis. ** P = 0.0054 (Mann-Whitney U Test). (B) Spearman Correlation between neutrophil counts and microbial diversity (left) or species richness (right) in the BAL. Shannon diversity and species richness are shown in the y-axis as a function of BAL neutrophil counts.

As neutrophils contribute to the production of reactive oxygen species during inflammation, which could modulate microbial communities on mucosal surfaces such as the lungs, we used neutrophil count in the BAL as a surrogate for lung inflammation. As expected, neutrophil count in the BAL of CF children was significantly higher compared to non-CF controls (Fig 7A). In non-CF controls, 6/7 (86%) with high neutrophil counts (>50 neutrophils/uL) had positive viral PCR, compared to 5/19 (26%) with low neutrophil counts (p<0.05). We observed a negative correlation between neutrophil counts in the BAL and microbial diversity, but not species richness (Fig 7B). However, no significant difference in BAL microbial diversity and richness was observed between children with and without prior antibiotic exposure (S2 Fig).

## Discussion

In this study, we examined the differences between upper and lower airway microbiome communities in cystic fibrosis children and non-CF disease controls by sampling the oropharynx and lungs concurrently using throat swabs and bronchoalveolar lavage. We also examined the correlation between airway inflammation and airway microbiome in CF patients. We found significant differences between upper and lower airway microbiome in both CF and non-CF patients in terms of microbial richness and diversity as well as the composition and structure of microbial communities (i.e. bacterial taxa). In both CF and non-CF subjects, lower airway microbiome displayed lower diversity and richness with a different bacterial composition compared to their upper airways. There is a dearth of studies comparing upper airway microbiota with lower airway microbiota in children using bronchoalveolar lavage for sampling of lower airways. Kloepfer et al. showed increased richness and diversity of BAL fluid microbiome compared to nasopharyngeal samples in children undergoing clinically indicated bronchoscopy [44]. In contrast, using expectorated and induced sputum as the sampling method for lower airways, Zemanick et al. showed differences between upper and lower airway microbiome similar to our study [29].

Differences between upper and lower airways in both CF and non-CF children could be attributed to the filtering effect of upper airway structures that decreases access of bacteria to lower airways and the effect of airway clearance mechanisms (i.e. cough and ciliary motility) which clears bacteria from the lower airways. In addition, higher innate immune effectors and antimicrobial proteins in the lower airways could also contribute to these differences. Regardless of the potential mechanisms, our data clearly demonstrate that lower airways constitute not only a more sterile environment but also a separate microbial environment from upper airways. Therefore, sampling of the upper airway in CF patients (especially children) for the purpose of understanding the lower airway microbiome is not supported by the present study. Numerous studies have used culture-based methods to compare upper airway to lower airway microbes and have focused mostly on assessing the accuracy (sensitivity and specificity) of throat swab or sputum cultures in detecting specific microorganisms in lower airways that are thought to be disease causing [31–33]. Most of these studies indicated a high level of accuracy and therefore provide a misleading impression of similarities between upper and lower airway microbial environments, which is not supported by our results using culture-independent techniques capable of capturing the entire microbiome spectrum.

Our results showed that both upper and lower airway microbiome clustered differently in CF subjects compared to that of non-CF controls, suggesting the possibility that CF airway disease is associated with a unique microbiome signature regardless of the observed differences between upper and lower airways microbiome. These differences between CF and non-CF patients do not appear to be directly related to antibiotic therapy but further studies are needed to confirm these results. Large longitudinal studies are also necessary in order to determine if airway microbiome not only differentiates between CF and non-CF subjects but also identifies different disease phenotypes and/or disease severity within the CF population. Our study was not powered to determine the correlation between disease severity as measured by lung function and airway microbiome composition. Previous studies, however, have suggested that progressive CF lung disease is associated with decreased richness and diversity of airway microbiome. Such decreased richness and diversity was most likely attributed to antibiotic treatment. To circumvent this major confounder, future studies would require analysis of CF patients at a younger age. The advent of newly approved CF disease modifying treatments using CFTR correctors and potentiators would also provide an opportunity to study the direct effect of CFTR dysfunction on airway microbiomes structure and composition.

Our 16S rRNA analysis demonstrates significant inter-individual variations in taxonomic profiles within both CF and non-CF microbiota (Fig 4). However, predicted functions encoded by the airway metagenome were more conserved within each airway habitat and within groups. For example, in both CF and non-CF controls, genes involved amino sugar and nucleotide sugar metabolism, fructose and mannose metabolism, galactose metabolism, starch and sucrose metabolism, and phosphotransferase system dominated the oropharyngeal metagenome compared to the lung metagenome (S1 Fig). The abundance of these genes likely reflects the energy requirement of the resident oropharyngeal bacteria in metabolizing carbohydrates. On the other hand, gene categories related to amino acid and fatty acid biosynthesis and metabolism were more abundant in the lung metagenome. The functional differences between upper and lower airway microbiota are consistent with our observation that upper and lower airway microbiome are distinct. Compared to non-CF controls, functions related to bacterial motility proteins, two-component system, flagella assembly, and secretion system were differentially enriched in both the oropharynx and lung metagenome of CF children (Fig 6), a feature consistent with the pathogenic potentials of typical organisms colonizing the CF respiratory tract. In contrast, genes involved in the synthesis and metabolism of nucleic acids and protein dominated the airway metagenome of non-CF controls (Fig 6), which likely reflects the basic requirements of resident microbes in the respiratory tract. Going forward, elucidating the role of key pathogenic bacteria encoding virulence-related functions and understanding the basis of individual variations in microbiomes and metagenomes in CF will be essential in future studies.

Using BAL neutrophil count as a surrogate marker of airway disease severity, we observed a negative correlation between neutrophil count and lower airway microbiome diversity in CF patients, suggesting that CF disease severity is associated with significant microbiome changes regardless of antibiotics treatment. Our data is consistent with recently published studies showing similar correlation [45–47]. We can only speculate that high neutrophil counts in lower airways disrupt microbial diversity and composition through its microbicidal effects including phagocytosis, neutrophil proteases and production of oxygen free radicals. Since neutrophils are the final effector cells in the cascades of airways immune response and since the innate immune function of CF airways is defective due to changes in mucus properties and pH of airway surface fluid, CFTR dysfunction leads to overstimulation of adaptive immune response and high neutrophil abundance. It is plausible that overabundance of neutrophils in severe CF disease eventually leads to a less diverse microbiome, but at a high price of inflammatory response that leads to airway damage. Future longitudinal studies are needed to determine if certain early changes in the pattern of lower airway microbiome can be predictive of rapidly progressive lung disease.

There are several limitations in this study. First, there was a significant difference in age range between our CF and non-CF cohorts. Because microbiome is known to vary with age from infancy to school age, this could play a role in the observed differences in diversity between groups. Second, our CF cohort was not homogeneous as BAL samples were obtained in some patients during antibiotic therapy. The number of CF children without prior antibiotic therapy in our cohort was too small to allow direct comparison with controls. However, we observed no significant difference in lung microbial diversity and richness between children with and without antibiotic exposure (S2 Fig). On the other hand, antibiotic exposure should have little impact on the comparison of upper versus lower airway microbiome within individuals. Finally, we used an *in-silico* approach (PICRUSt) to compare functions of CF and non-CF airway metagenomes. However, biological functions could not be determined using this approach and additional metagenomics or metatranscriptomics analyses are necessary to validate our findings.

In conclusion, the differences between upper and lower airway microbiome and their predicted gene functions highlight the limitations of using upper airway samples as a surrogate to study lower airway microbiome in CF patients. Our results suggest that lower airway microbiome diversity may serve as a marker of airway disease severity in CF. Going forward, airway microbiome may be valuable for identifying different CF disease phenotypes and/or predicting response of new CFTR modulators on disease course.

## Supporting information

S1 Fig Differentially abundant gene functions in (A) CF and (B) non-CF controls. Functional categories of genes of the airway metagenome were predicted using PICRUSt, and differentially abundant functions were then identified by using linear discriminant analysis (LDA) coupled with effect size measurements (LEfSe). Gene functions enriched in upper airway metagenome (sampled using throat swabs) are indicated with positive linear discriminant analysis scores (green), and functions differentially enriched in lower airway metagenome (sampled by BAL) are indicated with negative linear discriminant analysis scores (red).
(TIF)

S2 Fig Richness (left) and microbial diversity (right) in the BAL microbiome according to recent antibiotic exposure. (A) Number of OTUs (B) Shannon diversity, are shown in the y-axis, and subjects were grouped according to antibiotic exposure (yes or no) within the month prior to sampling. The means were compared using Mann-Whitney U test.
(TIF)
