# Supplementary figures and images for: Upper versus lower airway microbiome and metagenome in children with cystic fibrosis and their correlation with lung inflammation
Source: PLoS One. 2019 Sep 19;14(9):e0222323. doi: 10.1371/journal.pone.0222323 (PMC6752789; doi:10.1371/journal.pone.0222323)

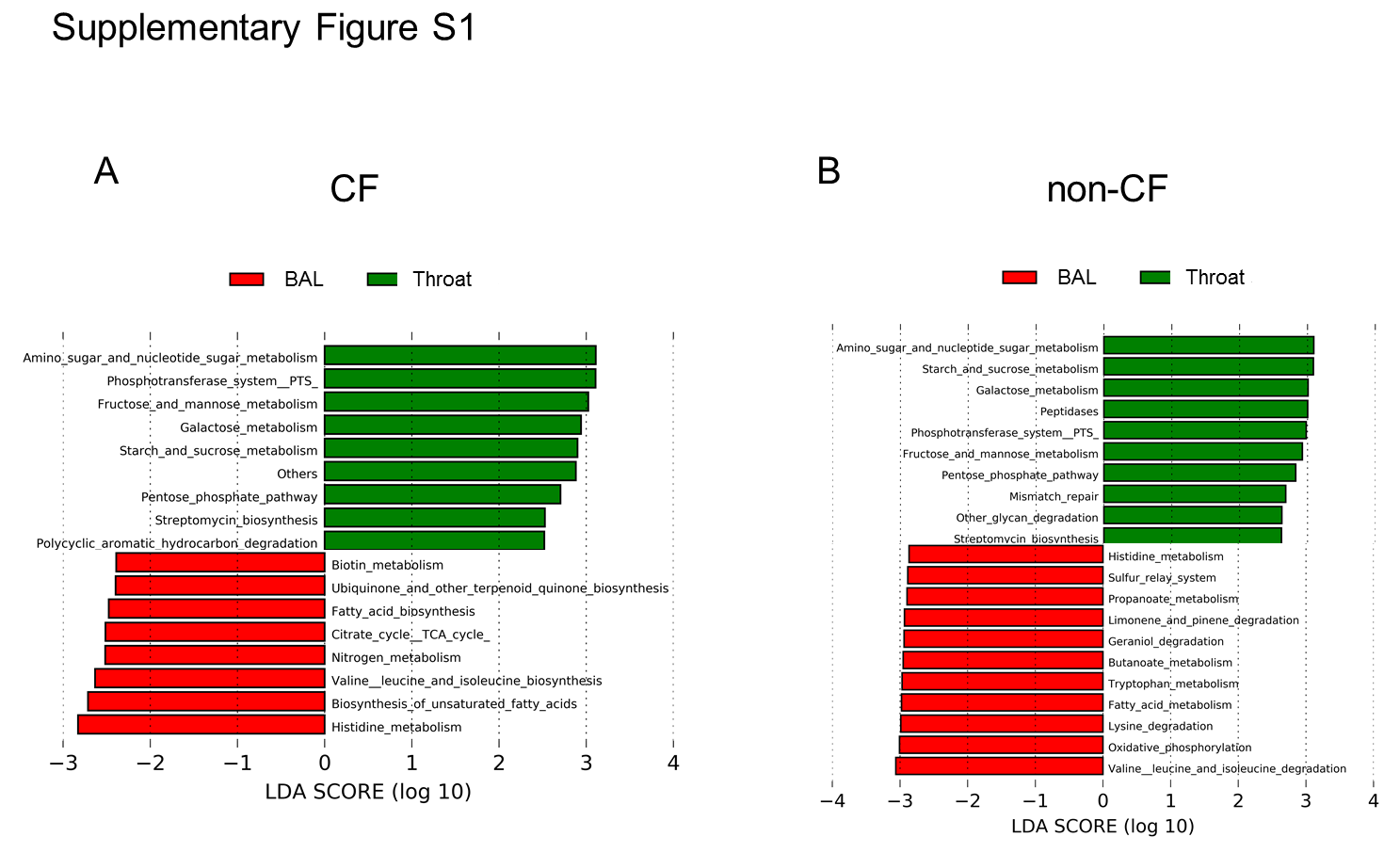

Supplement: S1 Fig — Differentially abundant gene functions in (A) CF and (B) non-CF controls. Functional categories of genes of the airway metagenome were predicted using PICRUSt, and differentially abundant functions were then identified by using linear discriminant analysis (LDA) coupled with effect size measurements (LEfSe). Gene functions enriched in upper airway metagenome (sampled using throat swabs) are indicated with positive linear discriminant analysis scores (green), and functions differentially enriched in lower airway metagenome (sampled by BAL) are indicated with negative linear discriminant analysis scores (red). (TIF) [file pone.0222323.s001.tif]

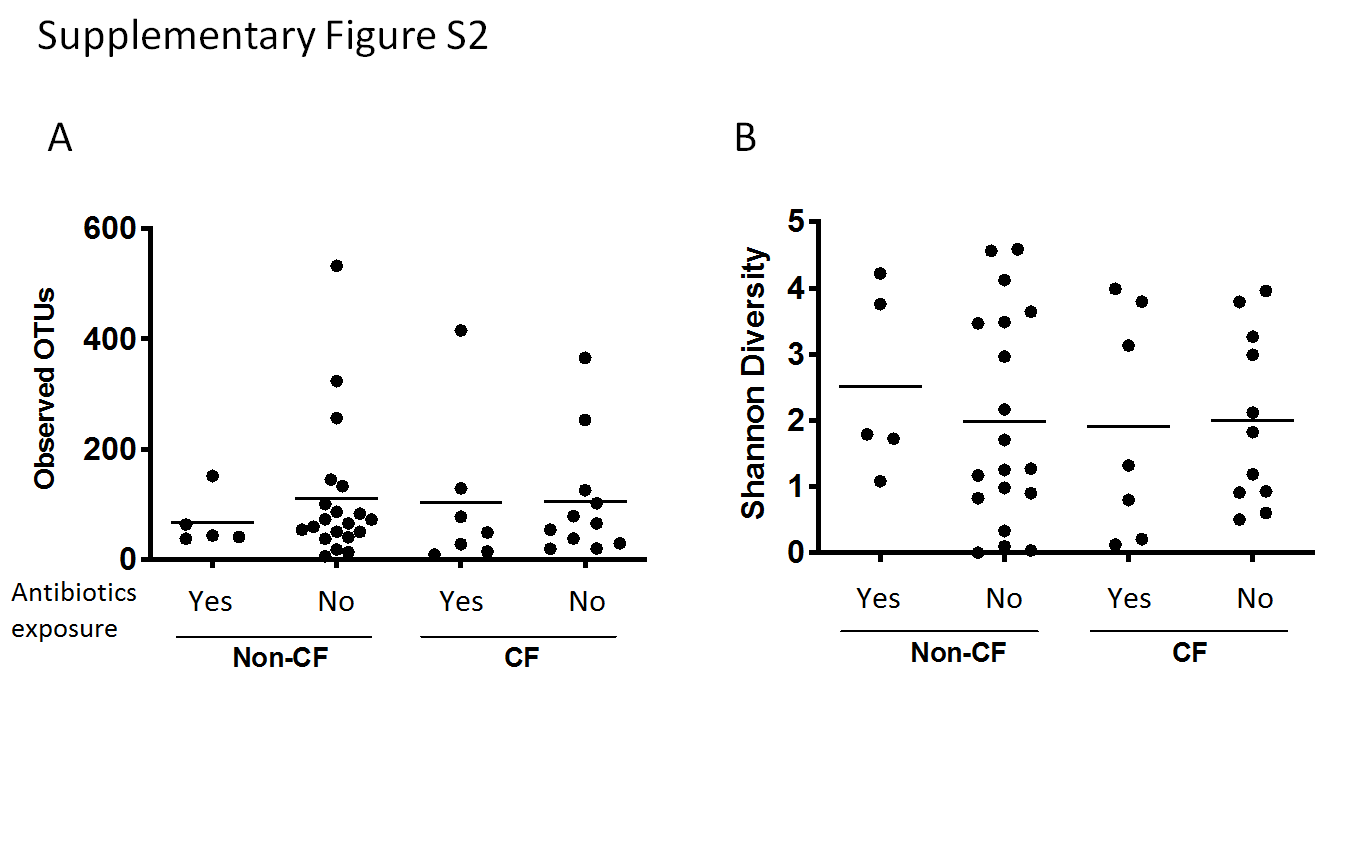

Supplement: S2 Fig — Richness (left) and microbial diversity (right) in the BAL microbiome according to recent antibiotic exposure. (A) Number of OTUs (B) Shannon diversity, are shown in the y-axis, and subjects were grouped according to antibiotic exposure (yes or no) within the month prior to sampling. The means were compared using Mann-Whitney U test. (TIF) [file pone.0222323.s002.tif]
